# Status of Malaria Vaccine Introduction—Africa, 2019–2024

**DOI:** 10.4269/ajtmh.26-0338

**Published:** 2026-09-17

**Authors:** Anh N. Ly, Jenny A. Walldorf, Mary J. Hamel, Eliane Pellaux-Furrer, Rafiq Nii Attoh Okine, Mgaywa G. M. D. Magafu, Jaymin C. Patel, Terri B. Hyde

**Affiliations:** ^1^ Epidemic Intelligence Service, U.S. Centers for Disease Control and Prevention, Atlanta, Georgia, USA;; ^2^ Global Immunization Division, U.S. Centers for Disease Control and Prevention, Atlanta, Georgia, USA;; ^3^ World Health Organization, Geneva, Switzerland

## Abstract

Malaria remains the primary cause of death among children younger than 5 years old in Africa. During 2019–2023, Ghana, Kenya, and Malawi piloted introduction of the first malaria vaccine, RTS,S/AS01, through the Malaria Vaccine Implementation Program. There are two malaria vaccines prequalified by the WHO: RTS,S/AS01 and R21/Matrix-M. The vaccines are recommended for children younger than 5 years old in endemic areas, prioritizing areas of moderate to high transmission. Data on vaccine introduction and doses shipped were obtained from the WHO and the United Nations Children’s Fund. As of December 2024, 17 countries in Africa had introduced the malaria vaccine with support from Gavi, the Vaccine Alliance, and another eight countries had approval for Gavi support. During 2023–2024, a total of 13,250,098 vaccine doses were shipped to countries. The introduction of the malaria vaccine presents an integration opportunity with preventive health services to reduce child morbidity and mortality.

## INTRODUCTION

Malaria causes substantial morbidity and mortality, with an estimated 263 million malaria cases and 597,000 malaria deaths globally in 2023.^1^ Countries in Africa account for 94% of cases and 95% of deaths, with approximately three quarters (76%) of malaria deaths occurring among children younger than 5 years old. The WHO’s Global Technical Strategy for Malaria 2016–2030 aims to reduce malaria incidence and mortality rate by 90% by 2030 compared with 2015 levels; however, since 2015, progress in reducing malaria has stalled.^2^ In addition to other interventions, malaria vaccines can now be deployed for malaria prevention. In 2019, introduction of the first approved malaria vaccine, RTS,S/AS01, was piloted in moderate- to high-transmission areas in Ghana, Kenya, and Malawi through the Malaria Vaccine Implementation Program (MVIP) coordinated by the WHO.^1^ Vaccine introduction resulted in a 13% reduction in all-cause mortality (excluding injury) and a 22% reduction in severe malaria hospitalization.^3^ This impact was reached during a period of vaccine scale-up when, on average, approximately 70% of children had received the first three doses of vaccine and less than 40% had received the fourth dose. In 2021, the WHO recommended RTS,S/AS01 for the prevention of *Plasmodium falciparum* malaria in children living in malaria-endemic areas, prioritizing moderate- to high-transmission areas.^4^ The RTS,S/AS01 malaria vaccine is recommended to be provided in a schedule of three doses at least 1 month apart starting from 5 months of age, with a fourth dose given in the second year of life to prolong protection. A fifth dose may be considered a year after the fourth dose in areas where malaria risk remains high after 3 years of age. Immunization programs may consider delivering the vaccine following an age-based, seasonal, or hybrid approach.^4^

Because of initial supply constraints of the RTS,S/AS01 vaccine, a framework was developed by the WHO to ensure prioritization of the vaccine among malaria-endemic countries.^5^ The framework was applied once in mid-2023 and resulted in 12 countries being able to start malaria vaccine implementation through a phased approach, with subnational introduction in areas with high child mortality rates and high *P. falciparum* malaria prevalence or incidence. The WHO recognized that reaching high vaccine coverage in these highest-need areas, where health systems are often weakest, would be challenging.^6^ The WHO recommendation of the R21/Matrix-M vaccine has resolved the constraints on malaria vaccine supply; the vaccine was prequalified by the WHO in late 2023.^1^ This was incorporated into the existing recommendation as a second-in-class RTS,S/AS01-like vaccine with comparable efficacy given on the same schedule. No preferential recommendation has been made between the two vaccines. The availability of two WHO-recommended malaria vaccines since late 2023 has resolved previous supply constraints. Countries that initially received limited vaccine doses have been able to apply to Gavi for expansion to additional geographic areas, covering up to 85% of the target population in moderate- to high-transmission areas.

## MATERIALS AND METHODS

This report examines malaria vaccine introduction during 2019–2024. The number of vaccine doses shipped to countries was obtained from the United Nations Children’s Fund (UNICEF) Immunization Market Dashboard.^7^ The status of malaria vaccine introduction by country was obtained from the Vaccine Introduction Status Dashboard.^8^ The percentage of children in the target population of the pilot countries receiving malaria vaccine was based on administrative data from Ministries of Health obtained from 2024 World Malaria Report.^1^ The vaccine introduction map was created using R v. 4.4.0 (R Foundation, Vienna, Austria).

## RESULTS

During 2019–2023, more than 6 million malaria vaccine doses were shipped by UNICEF to the three pilot countries, and the vaccines were administered through national childhood immunization programs. In 2023, a total of 2,709,700 doses were shipped, and in 2024, an additional 10,540,398 doses were shipped to countries. The proportion of children in the target population who had received one dose of the malaria vaccine among the pilot countries increased from 69% in 2019 to 84% in 2023, and the proportion of children who had received four doses increased from 6% in 2020 to 62% in 2023.^1^

**Figure 1.**
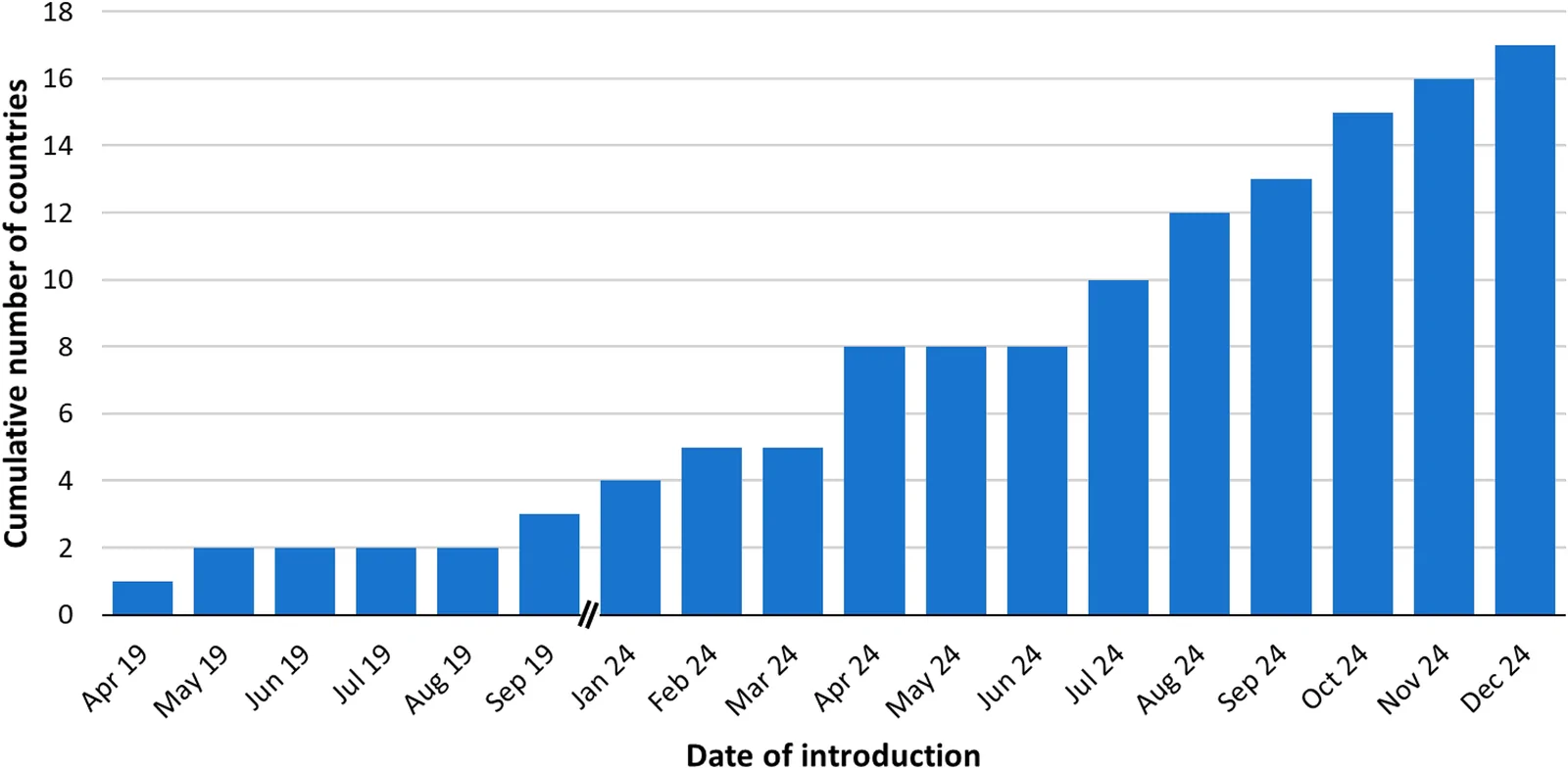
Dates of malaria vaccine introduction: 2019–2024.

**Figure 2.**
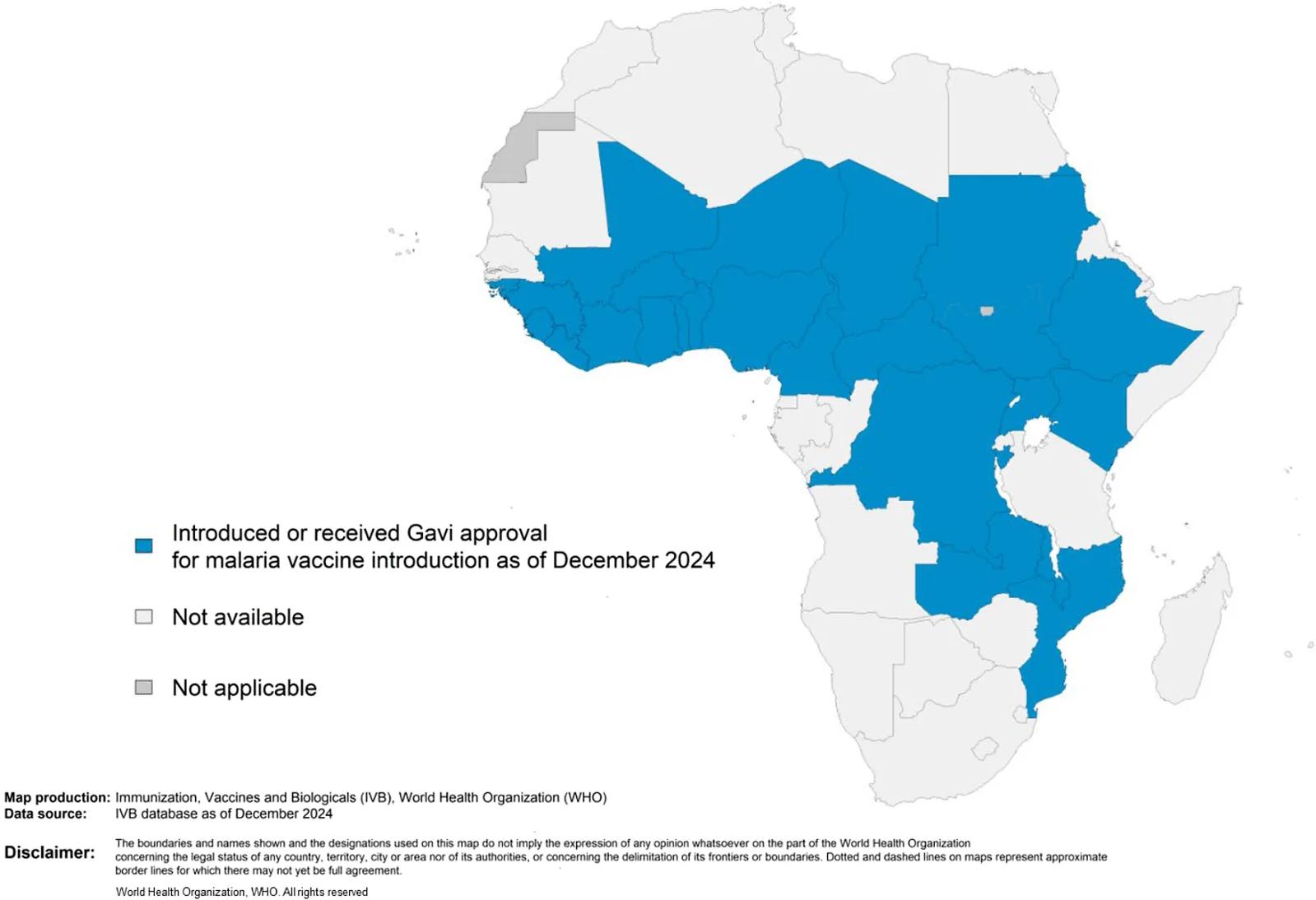
Status of malaria vaccine introduction by country: 2019–2024. Countries that have introduced the malaria vaccine as of December 2024 are Benin, Burkina Faso, Cameroon, Central African Republic, Chad, Côte d’Ivoire, Democratic Republic of the Congo, Ghana, Kenya, Liberia, Malawi, Mozambique, Niger, Nigeria, Sierra Leone, South Sudan, and Sudan. Countries that have received approval for malaria vaccine introduction support from Gavi are Burundi, Ethiopia, Guinea, Guinea-Bissau, Mali, Togo, Uganda, and Zambia.

In 2024, Ghana expanded vaccine introduction, and 14 additional countries introduced the vaccine into their routine immunization schedule subnationally with the support of Gavi, the Vaccine Alliance, starting with Cameroon in January 2024 (Figures 1 and 2). Of the 17 countries with malaria vaccine in their routine immunization schedule, 11 (65%) were classified as low income and the other 6 (35%) were classified as lower-middle income based on the 2024 World Bank Income Classification.^9^

Most countries have used an age-based approach, with the first three doses provided a minimum of 4 weeks apart and the fourth dose given in the second year of life. Flexibility in the schedule is allowed, and the WHO encourages countries to align the fourth dose with other second-year-of-life vaccines. Among countries that have introduced the malaria vaccine, the first dose is given at 5 months (6 countries) or 6 months (11 countries), and the fourth dose is given between 15 and 24 months of age (15 months: 6 countries; 16 months: 2 countries; 18 months: 6 countries; 22 months: 1 country; and 24 months: 2 countries).^8^ Two countries in seasonal transmission settings have elected to use the hybrid strategy.

An additional eight countries (six low income and two lower-middle income) received confirmation of Gavi support to introduce the vaccine in 2025 (Figure 2). Thirteen countries had received confirmation of Gavi support to scale up malaria vaccines and expand the target population. Central African Republic and Sierra Leone, where there is moderate or high transmission throughout the country, qualified for and received approval for Gavi support for a nationwide introduction.

## DISCUSSION

After the successful pilot introduction of the malaria vaccine through the MVIP, high demand for malaria vaccine is evidenced by the large number of countries that introduced the vaccine in the first 2 years since WHO prequalification (*n* = 14). Among the five countries reporting the highest number of malaria cases in 2023, three introduced the vaccine in 2024 (Nigeria, Democratic of the Congo, and Mozambique).^1^ As of March 2026, a total of 25 countries had introduced the vaccine.^10^

Challenges of implementing malaria vaccine are completing the multidose schedule and achieving high coverage for the fourth dose, particularly in the areas with weak health systems where the vaccine was initially introduced. A multicountry phase III trial from Africa indicated that the fourth dose was required to prolong protection; during a median of 48 months follow-up, vaccine efficacy against clinical malaria among children who received four doses was 36.3%, whereas vaccine efficacy against clinical malaria among those randomized to receive only three doses was 28.3%.^11^ In September 2025, the Strategic Advisory Group of Experts on Immunization and Malaria Policy Advisory Group reaffirmed the four-dose schedule for the RTS,S/AS01 malaria vaccine after reviewing a case–control study conducted in Ghana, Kenya, and Malawi.^12^ The study found that the four-dose schedule reduced severe malaria by 54% (95% CI: 39–66) and that the fourth dose provided an incremental effectiveness of 30% (95% CI: 9–45) compared with three doses. There was no evidence of rebound in severe malaria among children who missed the fourth dose; children who received only three doses from the age at which the fourth dose was due still showed sustained protection of 35% (95% CI: 15–50). During the pilot introductions, a survey conducted 30 months post introduction showed that coverage declined for subsequent doses (Ghana: 86% for dose 1 and 53% for dose 4; Kenya: 83% for dose 1 and 34% for dose 4; and Malawi: 74% for dose 1 and 33% for dose 4).^3^

Delivery of routine immunization during the second year of life when the fourth malaria vaccine dose is given has faced consistent challenges as seen with the lower uptake of the second dose of the measles-containing vaccine than the first dose of the measles-containing vaccine in many countries. Integration strengthening may improve uptake of the initial three malaria vaccine doses as well as uptake of the fourth dose of the malaria vaccine and other second-year-of-life vaccines. For instance, based on the experience from the MVIP and coverage at the 24-month time point, Ghana adjusted the timing of the fourth dose from 24 months to 18 months to coincide with the second dose of the measles and rubella vaccine, the meningococcal A conjugate vaccine, vitamin A supplementation, and growth-monitoring services.^13^^,^^14^ Despite the moderate uptake of the primary series and the low uptake for the fourth dose of the malaria vaccine, malaria vaccine introduction as part of the MVIP resulted in high impact, reducing all-cause mortality and hospitalizations for severe malaria.^3^

In addition to its contribution to child health and reduction of malaria illness and death, the introduction of the malaria vaccines provides an opportunity to strengthen the Expanded Program on Immunization. Malaria vaccine visits could facilitate engagement with children who have been out of the health system, and they provide opportunities for catch-up vaccination and strengthen the second-year-of-life platform. For example, introduction of the high-demand meningococcal serogroup A conjugate vaccine in Burkina Faso resulted in a 4.5% increase in coverage for the second dose of the measles-containing vaccine.^15^ The relationship between the national malaria and immunization programs provides an opportunity to integrate interventions to improve child health, such as home visits to provide seasonal malaria chemoprevention, insecticide-treated mosquito nets, caregiver education, or vaccine records review.

There are several limitations worth noting in this report. First, we did not include malaria vaccine coverage data for all countries as the countries not part of the MVIP were still in the early stages of vaccine introduction. Vaccination coverage is expected to increase and stabilize over time. Administrative data and representative coverage surveys will be used to document progress of the vaccine over time. Second, we did not have data on the number of malaria vaccine doses being administered. A comparison of the number of doses shipped to countries with the number of doses administered may be informative for monitoring the introduction and service delivery of the vaccines. Furthermore, community-based assessments can provide insights on vaccine demand among the population.

## CONCLUSION

Overall, malaria vaccination has been introduced in countries with moderate or high *P. falciparum* endemicity as part of comprehensive malaria control strategies, which could help accelerate progress toward the 2030 morbidity and mortality targets. The high demand for malaria vaccination can be leveraged to strengthen existing routine immunization programs. Opportunities to integrate malaria and immunization strategies in endemic countries could further reduce child mortality and improve child health.
